# Effects of atacicept on disease activity in patients with moderate to severe systemic lupus erythematosus: APRIL-SLE randomized trial

**DOI:** 10.1186/ar4669

**Published:** 2014-09-18

**Authors:** David Isenberg, Yong Li, Stephen Wax, David Wofsy, Caroline Gordon

**Affiliations:** 1 Centre for Rheumatology, University College London, UK; 2 R&D Global BioStatistics, EMD Serono Inc., Billerica, MA, USA; 3 Global Clinical Development Center, EMD Serono Inc., Rockland, MA, USA; 4 Rosalind Russell Medical Research Center for Arthritis, University of California, San Francisco, CA, USA; 5 School of Immunity and Infection, University of Birmingham, UK

## Background

Atacicept is a fusion protein that inhibits B-cell stimulating factors BLyS and APRIL, which are elevated in systemic lupus erythematosus (SLE). Atacicept 150 mg (A150) was associated with reduced BILAG A and B flares in the APRIL-SLE study. We examined efficacy of atacicept in preventing flares of SLE disease activity by the BILAG organ system and modified SELENA SLEDAI flare index and assessed corticosteroid use during a previously reported trial of atacicept versus placebo (PLC).

## Methods

Subjects with active SLE were treated with corticosteroid taper for 12 weeks. Subjects reaching BILAG C or D at weeks 10 and 12 were randomized at baseline 1:1:1 to receive PLC, atacicept 75 mg (A75) or A150 twice weekly every 4 weeks then weekly for 48 weeks. All patients received standard of care. Analysis was performed in the modified intention-to-treat population. BILAG and SELENA SLEDAI flares and corticosteroid usage were assessed 4 weekly.

## Results

The A150 arm was terminated early due to two fatal pulmonary infections. A lower proportion of subjects with >1 BILAG system A or B flare was observed in A150 and A75 versus PLC. Proportions of subjects with BILAG flare in each organ system were reduced in A150 versus PLC, except for vasculitis, which had similar proportions between groups. BILAG flares were most commonly observed in mucocutaneous and musculoskeletal systems. Proportions of subjects with severe SELENA SLEDAI flare during treatment in a *post-hoc *analysis were 19%, 11%, and 13% for PLC, A75, and A150, respectively. There was a dose-proportional decrease in the number of subjects who had at least one increase in steroid dose, and an increase of ≥20 mg/day (Table 1). Atacicept induced substantial reductions in Ig levels, which lessened during follow-up but did not fully return to baseline over 24 weeks. Atacicept treatment effect was observed in subjects with BLyS or APRIL levels ≥ median, but not < median. The difference was most pronounced in patients with baseline BLyS and APRIL ≥ median. Atacicept-treated patients with greatest absolute decrease in IgG and IgM experienced fewest new flares.

**Table 1:** Corticosteroid exposure post-randomization, modified intention-to-treat population

	Placebo	Atacicept 75 mg	Atacicept 150 mg
	**(*n *= 154)**	**(*n *= 157)**	**(*n *= 144)**

Zero dose increases, *n *(%)	108 (70.1)	115 (73.2)	122 (85.3)

	(*n *= 154)	(*n *= 157)	(*n *= 143)

Corticosteroids ≥20 mg/day, *n *(%)	43 (27.9)	39 (24.8)	17 (11.9)
Odds ratio		0.860	0.346
*P *value		0.563	0.001

## Conclusions

Reduction of the individual BILAG system and severe SELENA SLEDAI flares and corticosteroid use was associated with A150 treatment. Analysis of potential predictive biomarkers identified a subgroup of patients with higher BLyS and APRIL levels at baseline more likely to benefit from treatment. Further studies are required to clarify safety and efficacy of atacicept in SLE patients and associations between biomarkers and clinical response to atacicept.

## Trial registration

EudraCT: 2007-003698-13, NCT00624338

## Competing interests

DI, DW and CG are consultants for Merck Serono S.A. YL and SW are employees of EMD Serono Inc.

